# Determinants of Skeletal Muscle Preservation in Patients with Chronic Liver Disease

**DOI:** 10.31662/jmaj.2025-0292

**Published:** 2025-11-14

**Authors:** Masashi Aiba, Tatsunori Hanai, Kayoko Nishimura, Shinji Unome, Takao Miwa, Yuki Nakahata, Kenji Imai, Atsushi Suetsugu, Koji Takai, Masahito Shimizu

**Affiliations:** 1 Department of Gastroenterology/Internal Medicine, Gifu University Graduate School of Medicine, Gifu, Japan; 2 Center for Nutrition Support and Infection Control, Gifu University Hospital, Gifu, Japan; 3 Contributed equally

**Keywords:** branched-chain amino acids, liver cirrhosis, mortality, skeletal muscle mass preservation, sarcopenia

## Abstract

**Introduction:**

Preventing skeletal muscle mass loss may improve survival in patients with chronic liver disease (CLD); however, the clinical factors associated with maintaining skeletal muscle mass remain poorly understood.

**Methods:**

Clinically stable patients with CLD who underwent multiple computed tomography scans between March 2004 and April 2023 were enrolled. The annual rate of change in skeletal muscle area (ΔSMA/year) was assessed using a 3-dimensional image analysis system. Muscle mass preservation was defined as ΔSMA/year ≥0. The clinical factors associated with the prevention of muscle mass loss and their association with mortality were assessed using logistic regression analysis and Cox proportional hazards models.

**Results:**

Of the 586 patients (52% men; median age, 67 years; median model for end-stage liver disease score, 9), muscle mass was preserved in 124 (21%). Male sex (odds ratio [OR], 0.45; 95% confidence interval [CI], 0.27-0.73), alcohol-related liver disease (OR, 0.37; 95% CI, 0.18-0.77), and the branched-chain amino acids-to-tyrosine ratio (BTR) (OR, 1.17; 95% CI, 1.03-1.33) were independently associated with preservation of muscle mass. During a median follow-up of 3.7 years, patients with muscle preservation had a higher overall survival rate than those without (log-rank test, *P* = 0.007), with a hazard ratio (HR) of 0.58 (95% CI, 0.39-0.87). Preservation of muscle mass also independently predicted improved survival (HR, 0.56; 95% CI, 0.32-0.99).

**Conclusions:**

Male sex, alcohol-related liver disease etiology, and BTR are independent factors for muscle mass preservation, which is a significant determinant of survival in patients with CLD.

## Introduction

Sarcopenia, the progressive and generalized loss of skeletal muscle mass and strength associated with adverse outcomes, is a common complication in patients with chronic liver disease (CLD), particularly, in those with liver cirrhosis (LC) ^(1)^. Skeletal muscle mass typically decreases by 1% per year with age; however, in patients with LC, it decreases by 2% per year and patients with Child-Pugh C experience up to 6% muscle mass loss per year ^(2)^. CLD-related sarcopenia is associated with adverse outcomes, such as reduced quality of life, falls, fractures, infections, hepatic encephalopathy (HE), hepatic decompensation, and mortality before and after liver transplantation (LT) ^(1), (3), (4), (5), (6), (7)^. It is, therefore, imperative to identify patients with a rapid rate of skeletal muscle loss and those predisposed to sarcopenia at an early stage in the management of CLD ^(2), (8), (9)^. At the same time, it is also important to investigate the factors that can help prevent muscle loss and maintain muscle mass.

Several lines of evidence demonstrate that factors associated with sarcopenia include older age, male sex, lower body mass index (BMI), alcohol-related liver disease (ALD), and decreased liver function reserve ^(3), (10)^. These associations are especially apparent in patients with advanced LC ^(11), (12)^. On the other hand, nutritional interventions, such as branched-chain amino acid (BCAA) supplementation, exercise therapy, and a combination of both, may improve muscle mass, liver function reserve, and prognosis in patients with LC ^(5)^. In addition, improving sarcopenia to non-sarcopenia has been shown to reduce the risk of mortality in patients with LC ^(13)^. In view of these findings, identifying clinical parameters associated with preserving or increasing skeletal muscle mass in patients with CLD is a fundamental step toward developing treatment options for sarcopenia. However, few studies have focused on factors that may prevent or improve skeletal muscle loss in patients with CLD.

This study aimed to investigate the factors involved in the preservation of skeletal muscle mass in patients with CLD and to clarify whether the prevention of skeletal muscle loss improves the prognosis in these patients. The findings of this study may assist in developing effective strategies to preserve muscle mass and improve clinical outcomes in patients with CLD.

## Materials and Methods

### Study design, ethical considerations, and outcomes

This retrospective cohort study included patients with CLD without hepatocellular carcinoma (HCC) who underwent multiple computed tomography (CT) scans at least 3 months apart at Gifu University Hospital between March 2004 and April 2023. The patients underwent outpatient follow-up every 1-3 months according to the standard clinical practice guidelines for CLD ^(1), (14), (15), (16), (17), (18)^. All patients were followed up until death or LT. The overall survival was calculated from the first skeletal muscle evaluation until the occurrence of death, LT, last follow-up visit, or August 31, 2024.

The study was reviewed and approved by the Ethics Committee of Gifu University Graduate School of Medicine (approval number 2024-038), and the study protocol conformed to the ethical principles of the 1964 Declaration of Helsinki and its subsequent amendments. The primary outcome was to identify the factors associated with skeletal muscle preservation in patients with CLD. The secondary outcome was to determine whether inhibiting skeletal muscle loss had a beneficial effect on the prognosis in these patients.

### Study population

LC was diagnosed based on typical clinical manifestations, laboratory test results, non-invasive tests of fibrosis (Fib-4 index), liver imaging features, and histological findings when available ^(19)^. ALD was diagnosed in cases with a history of long-term excessive alcohol consumption (>50 g/day for women and 60 g/day for men) and no other identifiable cause of liver disease ^(20)^; all other cases were defined as non-ALD. Overt HE was diagnosed according to the West Haven criteria. The severity of liver dysfunction was determined using the model for end-stage liver disease (MELD) score ^(21)^.

The eligibility criteria were age ≥20 years and a diagnosis of clinically stable CLD of any etiology. Exclusion criteria included patients who refused to provide consent to participate in the study; any active malignancy, including HCC; pregnancy or lactation; history of organ transplantation; history of transjugular intrahepatic portosystemic shunt (TIPS) procedures; neurological, psychiatric, or orthopedic disorders that could result in a prolonged bedridden status; any condition that could result in prolonged malnutrition; and any life-threatening comorbidities, such as severe infection, cardiac failure, respiratory failure, or renal failure.

### Evaluation of body composition

The cross-sectional CT image areas at the third lumbar vertebra (L3) level of the skeletal muscle, subcutaneous adipose tissue, and visceral adipose tissue, which are all representative of the corresponding whole-body values, were evaluated using a 3-dimensional image analysis system (Synapse Vincent, Fujifilm, Tokyo, Japan), which accurately measured the boundaries of specific tissues in Hounsfield units (HUs) ^(22)^. The HU thresholds used were −29 to 150 HU for skeletal muscle, −190 to −30 HU for subcutaneous adipose tissue, and −150 to −50 HU for visceral adipose tissue. These areas (cm^2^) were then normalized by height (m^2^) to calculate the following indexes (cm^2^/m^2^): skeletal muscle index (SMI), subcutaneous adipose tissue index (SATI), and visceral adipose tissue index (VATI) ^(23)^. The annual rate of change in skeletal muscle area (ΔSMA/year) was calculated according to previous studies ^(2)^. The muscle preservation group was defined as ΔSMA/year ≥0, and the muscle loss group was defined as ΔSMA/year <0. The median time between the two CT scans was 2.3 years (interquartile range, 0.6-5.2 years).

### Data collection

Patient data on demographic characteristics and laboratory test results obtained within one month of the first CT scan were extracted from electronic medical records. Serum BCAA concentrations were assessed by using the enzymatic method (Diacolor BTR; Toyobo, Osaka, Japan). The number of patients receiving loop diuretics and BCAA supplementation was also examined because these may be associated with skeletal muscle changes ^(24)^. BCAA supplementation was defined as the use of oral BCAA granules (LIVACT; EA Pharma, Tokyo, Japan) and/or BCAA-enriched powder mix (Aminoleban EN; Otsuka Pharmaceutical, Tokyo, Japan) at the time of enrollment. BCAA supplementation was provided based on serum albumin levels, the Child-Pugh class, and sarcopenia status, in accordance with the guidelines for cirrhosis ^(14), (15)^.

### Statistical analyses

Continuous variables were presented as medians and interquartile ranges, and comparisons between the two groups were made using the Mann-Whitney *U* test. Categorical variables were presented as numbers and percentages, and comparisons between the two groups were made using the chi-square test or Fisher’s exact test. Survival probabilities were estimated using the Kaplan-Meier method, and comparisons between the two groups were performed using the log-rank test. The association between skeletal muscle preservation and its predictors was analyzed using univariate and multivariate logistic regression models, and the results were presented as odds ratios (ORs) with 95% confidence intervals (CIs). Prognostic factors for mortality were analyzed using univariate and multivariate Cox proportional hazards models, and the results were presented as hazard ratios (HRs) with 95% CIs. Multivariate logistic regression and Cox proportional hazards models were constructed based on variables of interest, such as cirrhosis etiology, BCAA-to-tyrosine ratio (BTR), muscle preservation, significant factors (p < 0.05) in the univariate models, and established risk factors in patients with CLD, such as age, sex, MELD score, ammonia, and loop diuretics. Covariate selection was predetermined to avoid overfitting, considering existing evidence, expert knowledge, and multicollinearity ^(25)^. Stepwise selection methods based on the Akaike information criterion and *P*-values were used for the logistic regression Cox proportional hazards models, respectively. All tests were two-tailed, and the statistical significance level was set at p < 0.05. Statistical analyses were performed using JMP Pro 17 software (SAS Institute Inc., Cary, NC, USA).

## Results

### Patient characteristics

The clinical characteristics of the enrolled patients are presented in Table 1. The study included 586 patients with CLD (307 [52%] men; median age, 67 years; median BMI, 23.0 kg/m^2^; median Fib-4 index, 5.48; median MELD score, 9). In the total cohort, 90% of the patients had LC, 73% had gastroesophageal varices, 41% had ascites, 6% had overt HE, 27% had diabetes mellitus, and 34% and 45% received loop diuretics and BCAA supplementation, respectively. The main etiology of CLD was hepatitis C virus (38%), followed by ALD (24%), hepatitis B virus (8%), autoimmune hepatitis (6%), metabolic dysfunction-associated steatohepatitis (6%), primary biliary cholangitis (6%), Wilson’s disease (1%), and cryptogenic liver disease (13%).

**Table 1.** Characteristics of the Study Population.

Characteristics	Total cohort	Muscle loss group	Muscle preservation group	*P* value*
	(n = 586)	(n = 462, 79%)	(n = 124, 21%)	
Age, years	67 (58-75)	66 (57-74)	69 (60-75)	0.281
Men	307 (52)	262 (57)	45 (36)	<0.001
Body mass index, kg/m^2^	23.0 (21.1-25.4)	23.0 (21.3-25.1)	22.5 (20.2-26.4)	0.806
SMI, cm^2^	43.4 (37.5-50.1)	44.3 (38.4-50.7)	39.1 (33.5-47.5)	<0.001
SATI, cm^2^	36.8 (21.1-57.2)	36.2 (21.0-56.3)	42.5 (24.0-73.3)	0.065
VATI, cm^2^	37.6 (21.3-56.3)	36.9 (21.8-55.1)	41.5 (18.5-61.1)	0.446
Diabetes	156 (27)	114 (25)	42 (34)	0.051
Liver cirrhosis	530 (90)	424 (92)	106 (86)	0.040
ALD cirrhosis	138 (24)	127 (28)	11 (9)	<0.001
Fib-4 index	5.48 (3.42-8.30)	6.03 (3.62-8.65)	4.17 (2.68-6.51)	<0.001
Gastroesophageal varices	429 (73)	348 (75)	81 (65)	0.030
Ascites	242 (41)	206 (45)	36 (29)	0.002
Overt HE	37 (6)	32 (7)	5 (4)	0.301
MELD score	9 (7-11)	9 (7-11)	8 (7-11)	0.012
Albumin, g/dL	3.2 (2.6-3.8)	3.2 (2.6-3.7)	3.5 (2.7-3.8)	0.060
Creatinine, mg/dL	0.72 (0.57-0.90)	0.72 (0.59-0.89)	0.72 (0.55-0.91)	0.693
Sodium, mEq/L	139 (137-141)	139 (137-141)	139 (138-141)	0.212
Total bilirubin, mg/dL	1.10 (0.80-1.70)	1.20 (0.80-1.70)	1.00 (0.70-1.42)	0.001
International normalized ratio	1.10 (1.02-1.24)	1.10 (1.03-1.25)	1.07 (1.00-1.17)	0.013
Ammonia, μg/dL	58 (42-86)	60 (42-89)	52 (39-73)	0.006
Zinc, μg/dL	69 (52-82)	69 (51-82)	71 (59-81)	0.284
BTR	4.1 (2.9-5.3)	3.9 (2.8-5.1)	4.5 (3.3-5.7)	0.007
BCAA supplementation	263 (45)	221 (48)	42 (34)	0.006
Loop diuretics	200 (34)	167 (36)	33 (27)	0.055

Data are presented as number (percentage) or median (interquartile range). *Continuous and categorical data were compared using the Kruskal-Wallis and chi-square tests, respectively. ALD: alcohol-related liver disease; BCAA: branched-chain amino acid; BTR: branched-chain amino acid-to-tyrosine ratio; HE: hepatic encephalopathy; MELD: model for end-stage liver disease; SATI: subcutaneous adipose tissue index; SMI: skeletal muscle index; VATI: visceral adipose tissue index.

**Figure 1.**
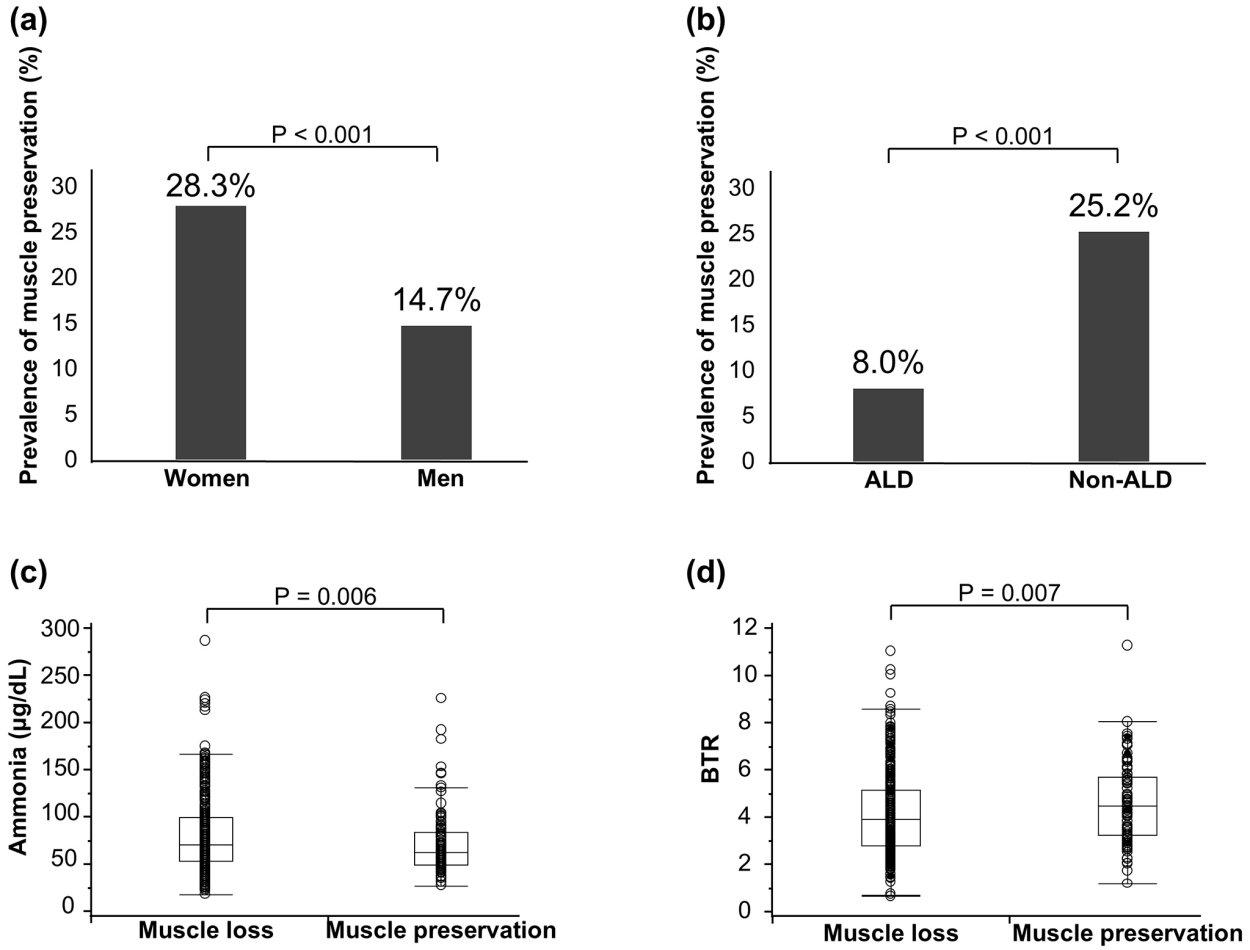
Factors associated with skeletal muscle preservation in patients with chronic liver disease. (a) Prevalence of muscle preservation between sexes, (b) prevalence of muscle preservation in patients with ALD and non-ALD, (c) comparison of ammonia levels between patients with and without muscle preservation, and (d) comparison of BTR between patients with and without muscle preservation. Data were analyzed using the Mann-Whitney *U* test and Pearson’s chi-square test. ALD: alcohol-related liver disease; BTR: branched-chain amino acid to tyrosine ratio.

A total of 124 patients (21%) were classified into the muscle preservation group and 462 (79%) were classified into the muscle loss group (Table 1). The muscle preservation group had a significantly higher proportion of female patients (Figure 1a, p < 0.001) and non-ALD etiology (Figure 1b, p < 0.001). The muscle preservation group also had significantly better liver function reserves (low rate of LC prevalence and LC-related complications and low Fib-4 index and MELD scores) than the muscle loss group (Table 1). In addition, the muscle preservation group had lower ammonia levels (Figure 1c, p = 0.006) and higher BTR levels than the muscle loss group (Figure 1d, p = 0.007). There were no significant differences between groups in terms of age, BMI, or diabetes mellitus.

### Predictors of skeletal muscle preservation in patients with CLD

In the univariate model, the predictors associated with muscle preservation included sex, SMI, SATI, diabetes mellitus, ALD, LC, Fib-4 index, gastroesophageal varices, ascites, MELD score, total bilirubin, international normalized ratio, ammonia, BTR, and the use of loop diuretics (all p < 0.05). The multivariate model showed that male sex (OR, 0.45; 95% CI, 0.27-0.73; p = 0.001), ALD (OR, 0.37; 95% CI, 0.18-0.77; p = 0.008), and BTR (OR, 1.17; 95% CI, 1.03-1.33; p = 0.016) were significantly associated with skeletal muscle preservation in patients with CLD (Table 2). The BTR remained statistically significant even when BCAA supplementation was included in the multivariate analysis.

**Table 2.** Predictors of Skeletal Muscle Preservation in the Multivariate Model.*

	OR (95% CI)	*P* value
Men vs. women	0.45 (0.27-0.73)	0.001
Diabetes	1.48 (0.90-2.44)	0.124
ALD vs. non-ALD	0.37 (0.18-0.77)	0.008
BTR	1.17 (1.03-1.33)	0.016

*Adjusted for age, sex, diabetes, ALD, liver cirrhosis, gastroesophageal varices, MELD score, ammonia, BTR, and loop diuretics. ALD: alcohol-related liver disease; BTR: branched-chain amino acid-to-tyrosine ratio; CI: confidence interval; MELD: model for end-stage liver disease; OR: odds ratio.

### Skeletal muscle preservation and overall survival in patients with CLD

**Figure 2.**
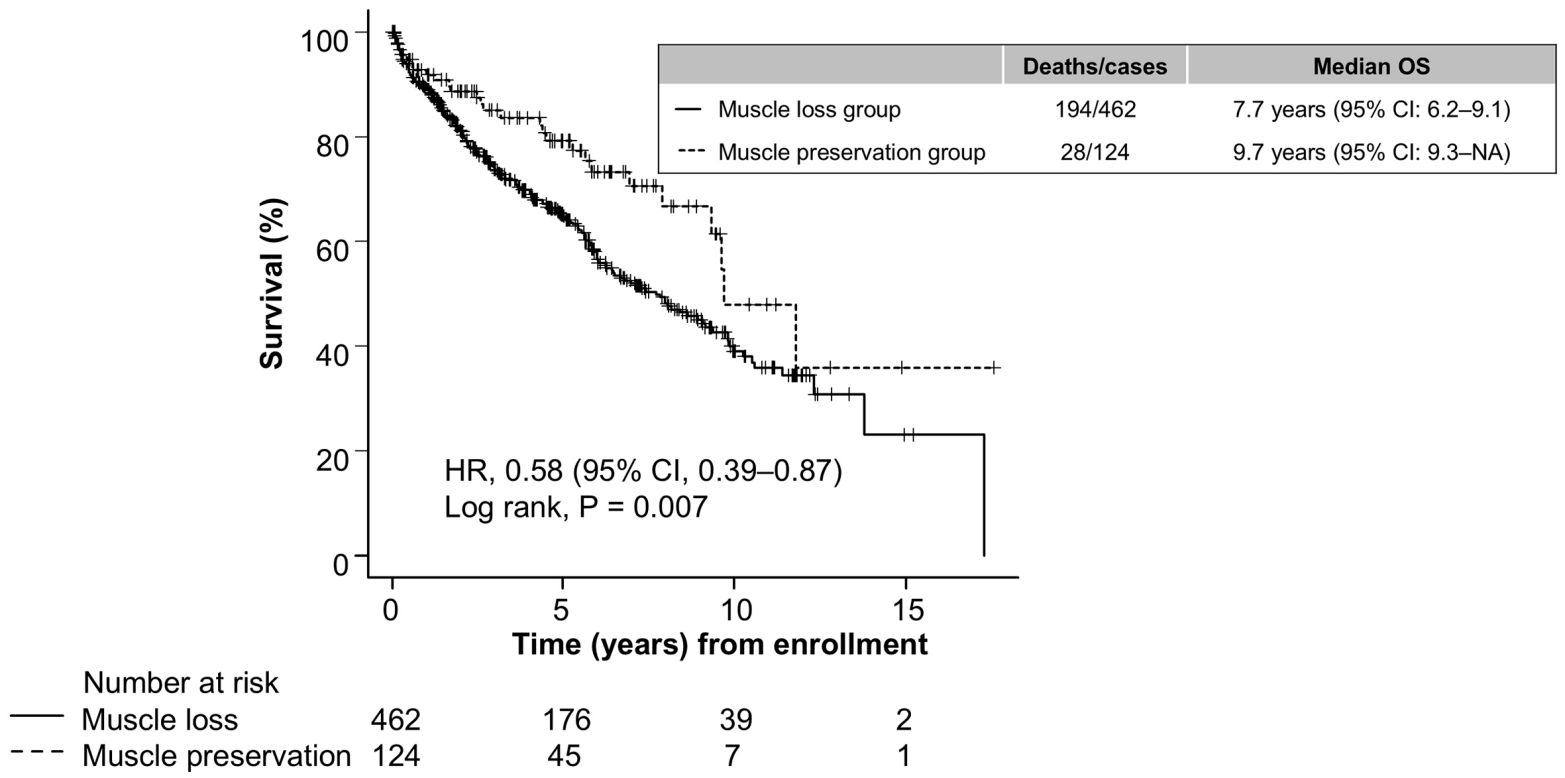
Survival curves for patients with chronic liver disease with and without preserved skeletal muscle mass. Survival over time was estimated using the Kaplan-Meier method, and the curves were compared using the log-rank test. CI: confidence interval; HR: hazard ratio; OS: overall survival.

Patients were followed up for a median period of 3.7 years (interquartile range, 1.4-6.4 years) until LT (n = 10), death (n = 212), or censoring (n = 364). Of the 212 patients who died, 136 died of liver failure, 32 of HCC, 13 of non-hepatic malignancy, five of infection, two of variceal bleeding, and 24 of other causes. In the muscle preservation group, 28 patients died and one received LT, with a median overall survival of 9.7 years (95% CI, 9.3-NA), whereas in the muscle loss group, 185 died and nine received LT, with a median overall survival of 7.7 years (95% CI, 6.2-9.1). Patients with muscle preservation had a lower risk of death than those without muscle preservation (Figure 2, p = 0.007), with an HR of 0.58 (95% CI, 0.39-0.87).

### Skeletal muscle preservation and mortality in patients with CLD

In the univariate model, the significant prognostic factors for mortality included skeletal muscle preservation, age, sex, SMI, SATI, VATI, LC, Fib-4 index, gastroesophageal varices, ascites, overt HE, MELD score, albumin, creatinine, sodium, total bilirubin, international normalized ratio, ammonia, zinc, BTR, and the use of loop diuretics (all p < 0.05). The multivariate model showed that skeletal muscle preservation (HR, 0.56; 95% CI, 0.32-0.99; p = 0.048), age (HR, 1.02; 95% CI, 1.01-1.04; p = 0.008), male sex (HR, 1.55; 95% CI, 1.06-2.27; p = 0.022), MELD score (HR, 1.10; 95% CI, 1.04-1.15; p < 0.001), and albumin (HR, 0.48; 95% CI, 0.36-0.64; p < 0.001) were significantly associated with mortality risk in patients with CLD (Table 3).

**Table 3.** Prognostic Factors for Mortality in the Multivariate Model.*

	HR (95% CI)	*P* value
Age, years	1.02 (1.01-1.04)	0.008
Men vs. women	1.55 (1.06-2.27)	0.022
MELD score	1.10 (1.04-1.15)	<0.001
Albumin, g/dL	0.48 (0.36-0.64)	<0.001
Skeletal muscle preservation	0.56 (0.32-0.99)	0.048

*Adjusted for age, sex, ALD, liver cirrhosis, MELD score, albumin, ammonia, zinc, BTR, loop diuretic use, and skeletal muscle gain. ALD: alcohol-related liver disease; BTR: branched-chain amino acid-to-tyrosine ratio; CI: confidence interval; HR: hazard ratio; MELD: model for end-stage liver disease.

## Discussion

Sarcopenia and rapid muscle wasting are strongly associated with poor outcomes in patients with CLD ^(2)^. Our recent study showed that ALD, cirrhosis, older age, male sex, and advanced liver disease are linked to rapid muscle wasting, leading to an increased risk of mortality, independent of established prognostic factors ^(12)^. Conversely, there is substantial evidence that an improvement in skeletal muscle contributes to a better prognosis in patients with CLD ^(13), (26), (27)^. Therefore, the identification of factors associated with muscle preservation represents an unmet clinical need for effective therapeutic interventions. The findings of the present study clearly showed that three factors―sex, liver disease etiology, and amino acid imbalance―were significantly associated with muscle preservation, which could potentially reduce the risk of death in patients with CLD by 44%. These findings have important implications for understanding the interventions that are likely to be more effective for specific patients.

One novel finding of this study is the importance of increased BTR in maintaining skeletal muscle mass. BTR, a nutritional indicator of LC, is determined by the plasma levels of BCAA ^(14), (15)^. BCAA, the cornerstone of nutritional therapy for LC, play an important role in muscle energy metabolism, promote protein synthesis, improve exercise performance, and have been shown to prevent sarcopenia and muscle wasting ^(28)^. A placebo-controlled trial reported that the addition of BCAA to standard treatment improved muscle mass in cirrhotic patients with sarcopenia ^(26)^. A 12-week combination of BCAA supplementation and exercise therapy improved muscle mass, strength, and functional exercise capacity in patients with cirrhosis ^(29)^, and this short-term intervention was associated with a reduced risk of hospitalization and mortality over a 3-year period ^(30)^. The results of the present study and these clinical trials strongly suggest that improving amino acid imbalance, such as through BCAA supplementation, may be useful for maintaining muscle mass in patients with CLD. Our findings are supported by previous studies, which demonstrated that amino acid imbalance are strongly associated with disease severity and long-term outcomes in the field of heart failure ^(31), (32), (33)^.

Another finding was that the male sex and ALD were associated with skeletal muscle loss in patients with CLD. In male patients with LC, serum testosterone, which has an anabolic effect on muscles, can decrease by up to 90% as liver function reserves deteriorate, contributing to sarcopenia in patients with cirrhosis ^(34), (35)^. A randomized controlled trial of testosterone therapy in male patients with LC and low testosterone levels reported an increase in muscle mass ^(36)^. However, the use of exogenous testosterone should be considered on an individual basis because it may be associated with an increased risk of HCC and thrombosis ^(1)^. The prevalence of sarcopenia in patients with ALD is 80%, which is higher than the prevalence of 10-40% in patients with CLD from other etiologies ^(20)^. Alcohol induces muscle autophagy, decreases the anabolic hormone insulin-like growth factor, and increases muscle ammonia levels, all of which lead to impaired protein synthesis and increased proteolysis, resulting in an increased risk of sarcopenia ^(1), (37)^. Alcohol-related cirrhosis results in muscle loss at double the rate compared with cirrhosis from other etiologies ^(8), (12)^, thus making abstinence from alcohol crucial for the preservation of skeletal muscle mass.

The present study shows that preservation of skeletal muscle mass reduces the risk of death in patients with CLD. The results support the findings of previous studies showing that preventing and improving sarcopenia contributes to the prognosis in patients with CLD ^(13), (27)^. Effective therapeutic strategies for improving sarcopenia in patients with CLD involve management of cirrhosis etiologies, including antiviral interventions and alcohol abstinence; the treatment of cirrhosis-related complications, such as ascites and portal hypertension; and the implementation of nutritional and exercise regimens ^(1), (6), (28)^. Because BTR is an independent factor associated with skeletal muscle preservation in patients with CLD, future studies should evaluate whether improving amino acid imbalance by nutritional therapy, including BCAA supplementation, can promote muscle protein synthesis and improve sarcopenia in these patients.

In addition to nutritional therapy, ammonia-lowering therapy is expected to provide a protective effect against skeletal muscle wasting ^(38), (39)^ because ammonia is a significant factor in the muscle wasting observed in patients with cirrhosis ^(40)^. Our study showed a clinically meaningful association between hyperammonemia (≥80 μg/dL) and muscle preservation, but it did not reach statistical significance in the multivariate logistic model. These findings may be partly explained by the fact that patients with hyperammonemia had already received significant ammonia-lowering therapies, including BCAA supplementation (p < 0.001), non-absorbable disaccharides (p < 0.001), nonabsorbable antibiotics (p < 0.001), and L-carnitine (p = 0.002), compared with those without hyperammonemia.

The strengths of our study are its large sample size (n = 586); detailed quantification of body composition using CT images, which is the gold standard for body composition assessment in LC; and the long-term follow-up period of 3.7 years. However, this study has several limitations. First, this was a retrospective longitudinal cohort study conducted at a single tertiary hospital; therefore, the generalizability of our results may be limited. Second, the retrospective nature of this study precludes the assessment of other variables, including dietary and exercise habits, environmental and socioeconomic factors, and accurate quantification duration of alcohol consumption ^(1), (6), (41)^. Third, although we defined muscle preservation as ΔSMA/year ≥0%, there is no established cut-off value to define muscle preservation. Finally, the treatment plan, including indications for BCAA supplementation and ammonia-lowering therapy, is largely at the discretion of the attending physician, which may introduce unintended bias. Therefore, our results should be interpreted with caution and validated through future large-scale clinical trials.

In conclusion, this study provides new and compelling evidence that skeletal muscle mass is preserved in approximately one-fifth of patients with CLD during the natural course of the disease. Sex, ALD, and BTR are strongly associated with muscle mass preservation, independent of age, comorbidities, and liver function reserve, which may contribute to an improved prognosis. Future studies should evaluate whether reducing alcohol consumption and improving BTR by nutritional therapy can improve muscle mass in patients with CLD.

## Article Information

### Acknowledgments

The authors would like to thank all medical professionals involved in this study.

### Author Contributions

All authors contributed to the conception and design of the study. Tatsunori Hanai, Kayoko Nishimura, Masashi Aiba, Shinji Unome, Takao Miwa, Yuki Nakahata, Kenji Imai, Atsushi Suetsugu, and Koji Takai collected and analyzed the data. The first draft of the manuscript was written by Tatsunori Hanai, and all authors commented on previous versions of the manuscript. All the authors have read and approved the final version of this manuscript. Masashi Aiba and Tatsunori Hanai contributed equally to this work.

### Conflicts of Interest

None

### Ethics Approval Statement

The study protocol was reviewed and approved by the Ethics Review Committee of the Graduate School of Medicine, Gifu University, Japan (approval No. 2024-038).

### Patient Consent Statement

Informed consent was obtained from all study participants using the opt-out method.

### Data Availability Statement

The data used in this study are available from the corresponding author upon reasonable request. However, additional approval from the Ethics Review Committee of the Graduate School of Medicine is required to share the data, in accordance with Japanese ethical guidelines.
